# Dissecting the actin cortex density and membrane-cortex distance in living cells by super-resolution microscopy

**DOI:** 10.1088/1361-6463/aa52a1

**Published:** 2017-01-11

**Authors:** M P Clausen, H Colin-York, F Schneider, C Eggeling, M Fritzsche

**Affiliations:** 1 MRC Human Immunology Unit, Weatherall Institute of Molecular Medicine, University of Oxford, Headley Way, OX3 9DS Oxford, UK; 2 Department of Physics, Chemistry, and Pharmacy, MEMPHYS—Center for Biomembrane Physics, University of Southern Denmark, Campusvej 55, 5230 Odense M, Denmark; 3 Kennedy Institute for Rheumatology, Roosevelt Drive, University of Oxford, Oxford OX3 7LF Oxford, UK; marco.fritzsche@rdm.ox.ac.uk

**Keywords:** super-resolution microscopy, actin cortex, membrane-cortex interface, plasma membrane

## Abstract

Nanoscale spacing between the plasma membrane and the underlying cortical actin cytoskeleton profoundly modulates cellular morphology, mechanics, and function. Measuring this distance has been a key challenge in cell biology. Current methods for dissecting the nanoscale spacing either limit themselves to complex survey design using fixed samples or rely on diffraction-limited fluorescence imaging whose spatial resolution is insufficient to quantify distances on the nanoscale. Using dual-color super-resolution STED (stimulated-emission-depletion) microscopy, we here overcome this challenge and accurately measure the density distribution of the cortical actin cytoskeleton and the distance between the actin cortex and the membrane in live Jurkat T-cells. We found an asymmetric cortical actin density distribution with a mean width of 230 (+105/−125) nm. The spatial distances measured between the maximum density peaks of the cortex and the membrane were bi-modally distributed with mean values of 50  ±  15 nm and 120  ±  40 nm, respectively. Taken together with the finite width of the cortex, our results suggest that in some regions the cortical actin is closer than 10 nm to the membrane and a maximum of 20 nm in others.

## 1. Introduction

**Figure 1.**
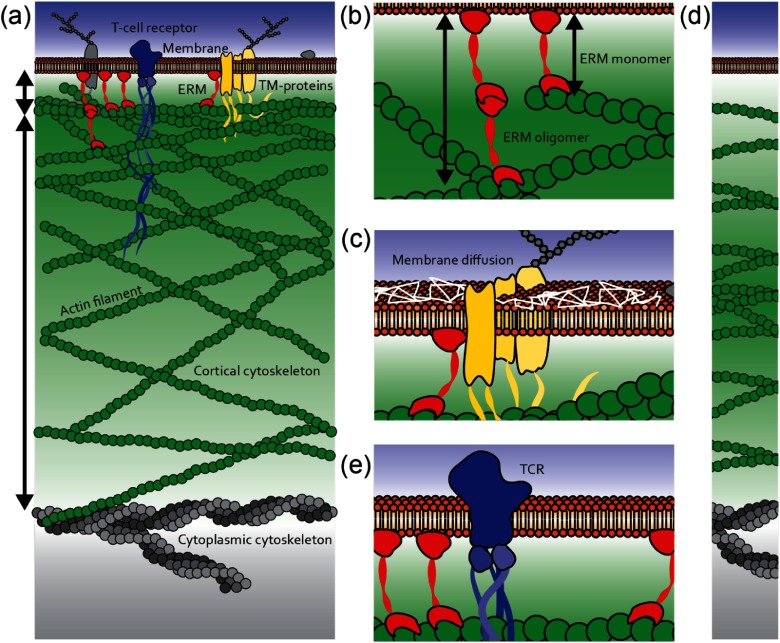
Overview of the membrane-cortex interface. (a) The membranous lipid bilayer (orange) is coupled via ERM proteins (red) to actin filaments (green) within the actin cortex. Cortical actin extends into the cell cytoplasm and is further interconnected with the cytoplasmic cytoskeleton (grey). Ligand receptors embedded within the membrane e.g. the T-cell receptor (TCR), associate with cytoplasmic protein binding partners (blue) such as its signaling domains that spans into the cortical actin network. Other transmembrane (TM) proteins (yellow) associates with ERM proteins and form barriers in the membrane plane. (b) Nanoscale spacing between the membrane and the inner boundary of the cortex may vary as a result of the formation of ERM oligomers. (c) Diffusion of membrane proteins and lipids is compartmentalized due to ERM associated transmembrane protein barriers. (d) The maximum density of the actin filament distribution within the cortex could also be thought to be largest in the centre of the cortex, in contrast to be the largest at the inner boundary next to the membrane as presented in (a). (e) The spatial distance between the membrane and cortex is particularly important in T-cell activation where triggering of the TCR result in association of several cytoplasmic proteins, some of which spans into the cortex restricting the mobility of the TRC complex.

Living cells continuously sample external stimuli and convert them into biological responses. Such cellular processes rely on the interactions of the cortical actin skeleton and the plasma membrane (figures 1(a)–(c)). External signals can be transmitted directly via specialized ligands such as the T-cell receptor expressed in the membrane of T cells, and/or indirectly via physical coupling through the membrane-cortex interface into the cell cytoplasm such as via mechano-transduction. Downstream the signals are then translated into various responses such as cell shape changes, spreading, differentiation, and migration [1–4]. Hence, success of signal transduction depends on the state of the membrane, the cortical actin cytoskeleton, and on the physical coupling of both of these layers. Specifically, the size of the interface, i.e. the spatial distance between those layers and the actin cortex density, is thought to underpin membrane and cortex homeostasis, resulting in altered cell morphology, mechanics, and function [5–7].

### 1.1. Cortical actin cytoskeleton and its density

Several lines of evidence suggest that the interface of the membrane and the cortex is directly regulated by the agency of the dynamic, filamentous scaffold known as the cytoskeleton. The cortical actin skeleton in particular, located beneath the plasma membrane, is thought to influence morphology, mechanics, and function due to its dynamic nature. It is a complex system, which has been estimated to be approximately 100–500 nm thick, that comprises polydispersed actin filaments undergoing continuous turnover with constant growth of the filaments at their barbed ends and shrinkage at their pointed ends [8, 9]. The actin kinetics determine cortex network structure whilst the dynamics of the filamentous structures govern the cell mechanics [10]. Joanny *et al* [11] recently derived the cortical actin density distribution at the membrane revealing two different density profiles for weakly and strongly active regimes of actin polymerization, suggesting the maximum actin density to be located close to the membrane periphery or further away, respectively (figure 1(d)). This theoretical prediction of the actin density has yet to be confirmed experimentally. One function of cortical actin could be to shield the cytoplasmic signaling domains of membrane-cortex ligands from the binding of signaling-associated molecules, precluding cell signaling (figure 1(e)) [12].

### 1.2. Membrane architecture and dynamics

Numerous membrane-dependent processes have been shown to be regulated by the membrane-cortex interface. The diffusion-reaction dynamics of membrane-cortex ligands, for instance of diffusing proteins and lipids and/or receptor binding to their extracellular agonists, has been demonstrated to affect initial signal detection, intracellular signaling, and signal translation into biological responses [6, 13, 14]. Membrane molecules usually do not freely diffuse but are transiently trapped or compartmentalized when interacting with immobilized or slow moving entities. Actin modulates lateral diffusion by inducing lipid and multi-protein assemblies and shifting their free diffusive behavior into trapped- and compartmentalized- or hop-diffusion (figure 1(c)) [6, 15–18]. Similarly, indirect signal transduction such as extracellular mechanical stress via the membrane-cortex interface is influenced by the same processes as discussed above. All of the described processes are undoubtedly influenced by two main characteristics of the actin cytoskeleton, the spacing between the membrane and the actin cortex, i.e. the membrane-cortex distance, and the density of the actin cortex.

### 1.3. Membrane-cortex distance

Nanoscale spacing of the membrane-cortex interface modulates the molecular activity and interactions of the membrane and cortex. The membrane is physically tethered to the underlying cortical network structure by proteins of the ERM family (ezrin, moesin, radixin) (figure 1(a)) [19].The kinetics of these membrane-cortex linkers, such as the association/dissociation rates of these molecules and the probability of the linkers to form oligomers, influences the binding of the membrane layer to actin [5]. The local concentration of individual linkers and number of oligomers per link thereby determine the local distance between both layers and adhesion force (figure 1(b)). Membrane-cortex adhesion measurements suggested a local force of 16 pN per link [20]. Low spatial membrane-cortex distances or less permeable cortices have a stronger influence on membrane hydrodynamics and produce more profound modifications of the membrane architecture [20]. Increasing membrane-cortex adhesion can be achieved via over-expression of constitutively active ezrin mutants such as ezrinT567A and results in decreased spacing of the membrane-cortex interface and induces high membrane tension and spherical morphology in HeLa cells [5, 21]. In contrast, over-expression of constitutively inactive ezrin mutants such as ezrinT567D decreases the adhesion energy and thus increases the spacing of the membrane-cortex interface, resulting in weakly defined cell boundaries [5, 21]. Similarly, mechano-transduction of extracellular mechanical force is altered upon variations in the nanoscale spacing between membrane and cortex [19, 22].

### 1.4. Determination of actin cortex density and membrane-cortex distance

Characterization of many of the above described processes at the interface of membrane and actin cortex could previously only be studied at a coarse-grained level because of limits in experimental observation techniques. Specifically, the ultra-structural characterization of actin networks has been hampered [9, 23]. One fundamental limitation has been the high actin density with small mesh-sizes of cortical structures [9, 24]. Typical mesh-sizes of 10–15 nm exclude the use of conventional optical microscopy, due to its limited spatial resolution, and so far only scanning-electron-microscopy and super-resolution STORM-based optical microscopy were able to visualize cortical mesh networks [25]. Nevertheless, these techniques have not been able to provide information about the network structure such as the cortical actin density or the molecular actin filament lengths because the high actin density precludes the accurate identification of filament ends [9]. Attempts to predict the spatial distance between the membrane and the cortex from the crystal structure of full-length ERM proteins such as ezrin and moesin were not successful. Crystallization of some of the domain such as dormant and active FERM domain of ezrin and moesin suggested spacings in the order of 10–50 nm for monomeric linkers [26–28]. A recent attempt to estimate the spatial distance between the membrane and the cortex from electron tomography reconstructions of flat cross-section samples in fixed PtK2 cells predicted  <10 nm [16]. At the same time, electron microscopy observations suggested that the cortex is uniformly dense, ends abruptly at the cytoplasm interface, and is in direct contact with the plasma membrane [29].

All of these measurements required cell fixation. However, measurements on fixed cells may suffer from artifacts such as cell shrinkage and altered architecture of the plasma membrane and actin networks [30, 31], therefore the determination of actin cortex density and membrane-cortex distance measurements would favorably be performed on living cells. Using conventional confocal microscopy, Clark *et al* [8] determined for the first time the thickness of the actin cortex in living cells to approximately ~190 nm. In order to establish these results despite the limited resolution of  >200 nm of their microscope, they employed dual-color labeling of the actin cortex and a theoretical description of cortex geometry. Specifically, they assumed a uniform actin density and a direct contact between the cortical layer and the plasma membrane. Yet, it would be important to approach the architecture of the actin cortex without such assumptions, since it is still elusive whether the cortex is in direct contact with the membrane or whether there is nanoscale spacing between the cortex and the membrane. Unfortunately, the diffraction-limited nature of the confocal experiments precludes directly resolving the cellular actin density distribution at the membrane and the nanoscale spacing between the cortex and the membrane.

Here, using dual color super-resolution STED microscopy, we overcome previous shortcomings and accurately localize the fluorescently labeled plasma membrane and the actin cortex. The increased spatial accuracy offered by STED microscopy allowed us to directly determine the density distribution of the cortical actin cytoskeleton beneath the membrane and to measure the nanoscale spacing between those two layers in living Jurkat T-cells. Intriguingly, we found a bimodal distribution of the nanoscale distances between the cortex and the membrane, suggesting that cells could dynamically tune their membrane architecture.

## 2. Materials and methods

### 2.1. Cell culture

Jurkat T-cells were cultured in sterile RPMI-1640 (Sigma Aldrich, UK) supplemented with 10% FCS (PAA), 2 mM L-Glutamine (Sigma Aldrich), 1 mM Sodium Pyruvate (Sigma Aldrich), 10 mM HEPES (Sigma Aldrich), and 1% Penicillin-Streptomycin-Neomycin solution (Sigma Aldrich). Cells were maintained at 37 °C and 5% CO_2_ during culturing, and handling was performed in HEPA-filtered microbiological safety cabinets. Typically, cells were kept at a density between 5–9  ×  10^5^ cells ml^−1^.

### 2.2. Plasmid

The vector encoding human *β*-actin N-terminally tagged with mCitrine and SNAP (New England Biolabs, UK) were generated by amplification of the ACTB gene by PCR using oligonucleotide primers with cDNA from human embryonic kidney (HEK)-293 T cells as template. The product contained ACTB flanked by 5′ BamHI and 3′ NotI restriction nuclease sites and followed by a TAG STOP codon. Following digestion with BamHI and NotI, this was ligated into a pHR-SIN lentiviral expression vector containing the mCitrine or the SNAP gene upstream of the BamHI site in the correct reading frame. Sequence integrity was confirmed by reversible terminator base sequencing.

To obtain vectors C-terminally tagged encoding LifeAct-Citrine (New England Biolabs, UK) the gene were amplified by PCR to produce dsDNA fragments encoding LifeAct sequence followed by a Gly-Ser linker and flanked by 5′ MluI and 3′ BamHI restriction nuclease sites. Following digestion with MluI and BamHI, this was ligated into pHR-SIN lentiviral expression vectors containing the mCitrine gene downstream of the BamHI site in the correct reading frame. Sequence integrity was confirmed by reversible terminator base sequencing.

### 2.3. Generation of stable cell lines

Jurkat derived T-cell line stably expressing either LifeAct-Citrine or LifeAct-SNAP were generated using a lentiviral transduction strategy. HEK-293T cells were plated in 6-well plates at 3  ×  10^5^ cells ml^−1^, 2 ml/well in DMEM (Sigma Aldrich)  +  10% FCS (PAA). Cells were incubated at 37 °C and 5% CO_2_ for 24 h before transfection with 0.5 *µ*g/well each of the lentiviral packaging vectors p8.91 and pMD.G and the relevant pHR-SIN lentiviral expression vector using GeneJuice^®^ (Merck Millipore, UK) as per the manufacturer’s instructions. 48 h post transfection, the cell supernatant was harvested and filtered using a 0.45 *µ*m Millex^®^-GP syringe filter unit to remove detached HEK-293T cells. 3 ml of this virus-containing medium was added to 1.5  ×  10^6^ Jurkat T-cells in 3 ml supplemented RPMI-1640 medium. After 48 h, cells were moved into 10 ml full medium RPMI-1640 and passaged as normal.

### 2.4. Microscope coverslip preparation

Microscope coverslips (#1.5) were washed 3  ×  1 ml PBS before use. Prior experimentation, the coverslipes were hydrated with 1 ml Leibovitz L-15 medium (Londa, UK) and incubated at 37 °C and 5% CO_2_ for 15 min. Five minutes before imaging, Jurkat T-cells were pipetted onto the coverslipes and incubated for 5 min to allow the cells attaching to the surface.

### 2.5. Fluorescence dye labeling

SNAP-Cell TMR Star labeling was performed following the manufacturer’s guidelines (www.neb.com/products/s9105-snap-cell-tmr-star). The TMR dye was purchased from New England BioLabs, UK. Prior experimentation, cells were spun down at 1500 rpm for 3 min and re-suspeneded in 200 ml L15 medium with 1 *µ*M SNAP-Cell TMR Star. This labeling solution was then incubated at 37 °C for 30 min, 3 times washed in L15, and then incubated for additional 30 min. Finally, the cells were washed in L15 medium and re-suspended into 200 *µ*l L15 imaging medium.

### 2.6. Lipid membrane labeling

Fluorescent lipid analogs DPPE-Atto488 and DPPE-Atto565 were purchased from Atto-tec (Siegen, Germany). Cells were labeled by adding 1 *µ*l of 1 *µ*M lipid analog (in EtOH) to 1 ml of cell suspension for 15 min at 37 °C.

### 2.7. Calibration of the STED microscopy

The effective size of the STED microscope’s PSF was determined using a fluorescent microspheres (FluoSpheres, yellow–green (505/515), diameter 20 nm, Invitrogen, USA). This sample was prepared by diluting the beads in Milli-Q-water with dilution factor 1:10 000. A drop of diluted beads was attached to the coverslide using poly-L-lysine (Sigma Aldrich) and then the coverslip was mounted on a microscope slide and embedded in the mounting medium mowiol.

### 2.8. STED microscopy

STED microscopy experiments were performed on a Leica TCS SP8 3X microscope (Leica, Mannheim, Germany). The microscope was equipped with a pulsed super-continuum white-light laser (WLL, Koheras SuperK, 80 MHz) for excitation, and 592 nm 1.5 W and 660 nm CW STED lasers. Citrine and DPPE-Atto488 were excited at 488 nm, the 592 nm STED laser was employed, and fluorescence emission was collected at around 530 nm and 520 nm, respectively. SNAP-Cell TMR-Star and DPPE-Atto565 were excited at 555 nm and 565 nm, respectively, the 660 nm STED laser was employed, and fluorescence was collected at around 580 nm and 590 nm, respectively. For STED imaging, excitation laser intensities of ~1–5 *µ*W (measured directly at the focal plane) of the white-light-laser and ~60–212 mW (measured directly at the focal plane) of the 592 nm and 660 nm STED lasers were utilized to obtain a strong enough fluorescence signal as well as sufficient improvement in spatial resolution. Images were acquired at 1–5 s intervals to minimize loss of fluorescence due to photo-bleaching as well as cell phototoxic effects (of which we did not observe any in the recordings). All images were acquired on the Leica HyD detectors using time-gated detection. The dual-color images were acquired sequentially in the order 555 or 565 nm followed by 488 nm to avoid fluorophore damage and crosstalk of the 592 nm STED laser into the 555 nm or 565 nm detection. All live-cell experiments were done at 37 °C and 5% CO_2_.

For the analysis of the data, using the Huygens STED-Deconvolution-Wizard (Huygens Software, Netherlands; https://svi.nl/Huygens Deconvolution), only a moderate degree of deconvolution was applied to the recorded STED images to avoid deconvolution artifacts. The microscope’s point-spread-function (PSF) was directly calculated from the Leica imaging files, following standardized Huygens software guidelines (www.leica-microsystems.com/science-lab/huygens-sted-deconvolution-quick-guide/).

### 2.9. Confocal microscopy

Confocal microscopy experiments were performed on a Leica TCS SP8 3X microscope (Leica, Mannheim, Germany), as for the STED experiments without using the STED laser. All live-cell experiments were done at 37 °C and 5% CO_2_. For further details see section 2.8.

### 2.10. Computation of the membrane-cortex density distributions

Prior determination of the cortical actin density, the two color fluorescence images were deconvolved using Huygens Deconvolution Software (see sections 2 and 2.8). The cortex density was computed from the fluorescence images using custom-written MATLAB routines. The algorithm locates the membrane-cortex region (dashes-white-line in figure 4(b)) for each dual color cell image including the DPPE-Atto-565 (membrane channel) and the LifeAct-Citrine (F-actin channel) using a steerable ridge detection algorithm (http://bigwww.epfl.ch/demo/steerable/ index.html) [32]. For a predefined subset of pixels along the dashed-white-line defining the edge of the cell, the line intensity profile normal (solid-red-line) to the dashed-white-line was extracted for both the membrane and cortex fluorescence channels. To avoid the loss of asymmetric details of these line profiles during the analysis, they were separated into two groups depending on the location of the profiles left or right oriented from the vertical axes of each cell, realigned in space to their maximum peak intensity, and then normalized to the integral of each fluorescence intensity. Finally, we average all actin density distributions and plot them as presented in figure 4.

### 2.11. Computation of the nanoscale membrane-cortex distance

Prior computation of the nanoscale membrane-cortex distance, the dual color fluorescence images were deconvolved using Huygens Deconvolution Software (see sections 2 and 2.8). The spatial distance $|{{r}_{\text{c}}}-{{r}_{\text{m}}}|$ between cortex and membrane was computed from the fluorescence images using custom-written MATLAB routines. The algorithm locates the membrane-cortex region as described in the section ‘Computation of the membrane-cortex density distributions’. Finally, the geometric mean and the standard deviation were computed for both the normalized membrane and the cortex density distributions *ρ*_norm_.

Next, each density distribution for both the membrane and the actin cortex were fitted with a linear combination of a decaying Exponential to describe the background and of a Lorentzian function to locate the peak coordinates in the membrane and cortex profiles. Specifically, the density distributions were fitted with ${{\rho}_{\text{norm}}}=\alpha {{\text{e}}^{-\beta r}}+\frac{\gamma}{{{\left(r-\delta \right)}^{2}}+\epsilon}+\xi $, whereas *α* and *γ* are the amplitudes of the Exponential and the Lorentzian functions, respectively. The coefficient *β* is the exponential decay rate and *ξ* accounts for the offset of both functions. The parameter *δ* reports on the location of the function peak (membrane *r*_m_ and cortex *r*_c_) and *ε* on the corresponding peak width.

The spatial distance $|{{r}_{\text{c}}}-{{r}_{\text{m}}}|$ between cortex and membrane was computed from the geometric difference of the fitting peak location for the membrane *r*_m_ and cortex *r*_c_. In addition to these distance measurements between the maximum peaks of the membrane and the cortex, the spatial distance $\left|r_{\text{c}}^{\prime}-{{r}_{\text{m}}}\right|$ was computed between the closest boundary of the cortex at the location $r_{\text{c}}^{\prime}$ and of the membrane at location *r*_m_. The corresponding closest boundary was determined at the point of intersection of the actin density distribution and the membrane.

### 2.12. Statistical analysis

Standard student *t*-tests were performed for statistical comparisons of the membrane-cortex distance measurements. Experimental conditions were considered significantly different for *p*-values smaller than *p*  <  0.01. In total the actin density and spatial distance measurements were computed from *n*  =  1000–2000 single measurements per cell for *N*  =  30 cells.

## 3. Results

### 3.1. Dual color STED imaging of the membrane and actin cortex

**Figure 2.**
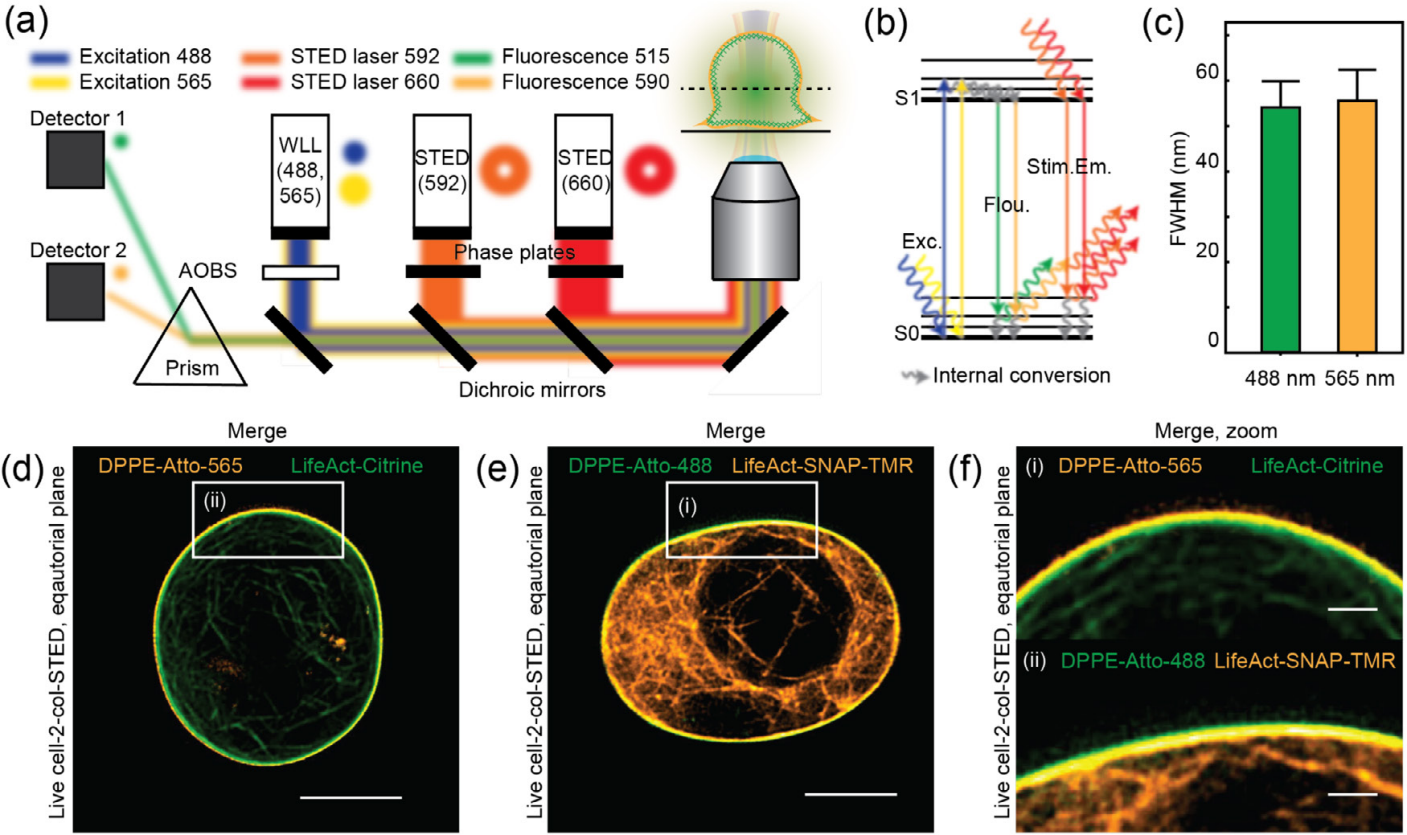
Schematic of the experimental STED setup. (a) Excitation light (488 nm (blue) and 565 nm (yellow), respectively) from a white light laser (WLL) was superimposed with donut-shaped STED light (592 nm (orange) and 660 nm (red), respectively), and focused onto the sample. The emitted fluorescence (515 nm (green) and 590 nm (light orange)) was selected via a prism and collected with detectors. (b) Fluorophores can be excited from the ground state (S0) to the first excited state (S1) by absorption of a photon. From the excited state, they can return to the ground state by emission of spontaneous fluorescence or emission depletion can be stimulated if the fluorophore absorbs a second photon resulting in decay to emission of a photon with the same wavelength. (c) The full-width-of-half-max (FWHM) of fluorescent beads was measured at 488 nm (STED 592 nm) and 565 nm (STED 660 nm) and used as the resolution of the setup. (d) and (e) 2-color STED images of live cells with the plasma membrane and actin cytoskeleton labeled. (d) The membrane was labeled with DPPE-Atto565 (orange), and the cortex with LifeAct-Citrine (green). (e) The membrane was labeled with DPPE-Atto488 (green), and the cortex with LifeAct-SNAP-TMR Star (orange). (f) Zoom-in of images in (d) and (e). Scale bars are 5 and 1 *µ*m.

To quantify the density distribution of cortical actin and the nanoscale spacing of the membrane-cortex interface, we performed experiments in live Jurkat T-cells using dual color super-resolution STED microscopy. For an optimal fluorescence labeling strategy, we generated a Jurkat T-cell line constitutively expressing LifeAct-Citrine (or Lifeact-SNAP) that targets solely the filamentous pool but not the pool of free monomeric actin, yielding distinct localizations of the cortical actin cytoskeleton beneath the membrane and the cytoplasmic actin cytoskeleton within the cell volume. Complementary to this, we used the fluorescent DPPE lipid analog DPPE (DPPE-Atto565 or DPPE-Atto488) for precise localization of the membrane. The STED microscopy setup (figures 2(a) and (b)) consisted of two channels, green and orange, with laser excitation lines at 488 nm (shown in blue) and 565 nm (shown in yellow), collection of fluorescence emission light at 515 nm (green) and 590 nm (orange), and STED lasers at 592 nm and 660 nm, respectively. The spatial resolution of the STED microscope was 50 nm  ±  10 nm for both the 592 nm and 660 nm STED lasers, as determined from images of standard 20 nm large fluorescent bead samples (figure 2(c)). This value estimates the best achievable spatial resolution of the optical microscopy system at minimal fluorescence background.

Using scanning confocal-based microscopy, most accurate measurements of the nanoscale spacing between membrane and cortex were achieved in the geometric center-plane of the cells, the so-called equatorial plane, half-way through the cell volume in the direction vertically away from the glass surface at which the cells were rested. Equatorial measurements provided a laterally-separated geometry of the membrane-cortex interface under which the STED microscope provided maximal spatial resolution (In contrast, at the basal plane at the bottom of the cells, closest to the glass surface, imaging would rely on the spatial resolution along the axial direction, which only equals the confocal resolution in the case of 2D-STED (~1 *µ*m) and ~100 nm for 3D-STED [33, 34]).

The largest bias in the determination of the membrane-cortex distance may stem from chromatic aberrations between the green and orange channels and potential labeling artefacts. Chromatic shifts would lead to de-localization of the membrane and actin cortex layers relative to each other in the dual-color images and would therefore result in erroneous distance quantifications. As a control, we exchanged colors for labeling the two layers: LifeAct-Citrine (green) and DPPE-Atto565 (orange) or LifeAct-SNAP tagged with the SNAP-dye TMR-Star (orange) and DPPE-Atto488 (green) for marking the actin cortex and membrane, respectively. Consistently, we found that the membrane was located at the outer boundary and the cortical actin network located at the inner boundary (figures 2(d)–(f)), and that quantification of actin-cortex density and membrane-cortex distance gave similar results (see below), suggesting that there was no statistical difference between the two labeling strategies, i.e. there was negligible bias by chromatic aberrations and the results were fluorescent probe independent (at least between the tested cases). In the following, we used the LifeAct-Citrine and DPPE-Atto-565 labeling strategy, mainly for practical reasons, since the alternative strategy yielded slightly lower signal to noise ratios in the final images of the membrane, and we also avoided additional stress for the T cells at the centrifuge spinning step during the SNAP-labeling with the soluble SNAP-TMR dye.

### 3.2. Quantification of the membrane and actin density distributions

**Figure 3.**
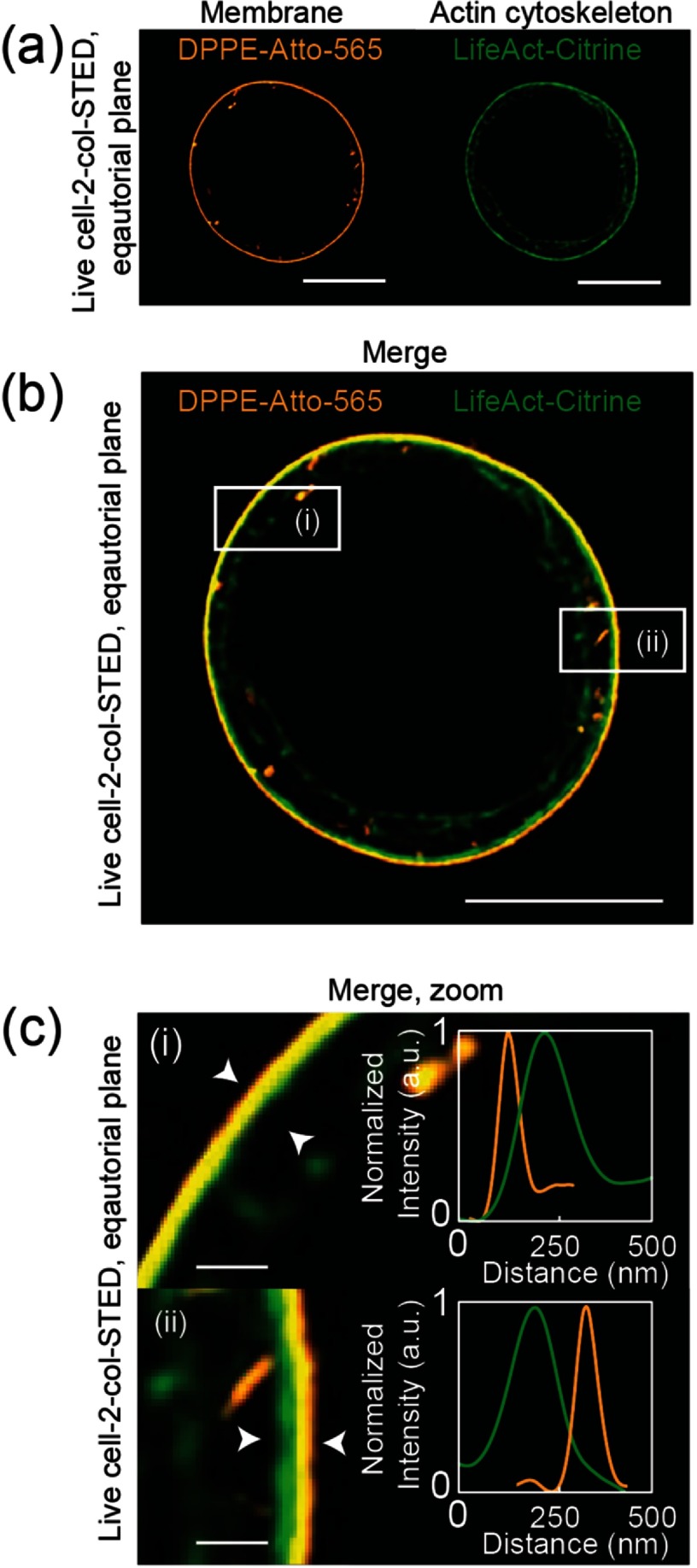
Quantification of the membrane-cortex interface using 2-color STED images of live Jurkat T-cells. (a) Representative images of the lipid membrane bilayer fluorescently tagged with DPPE-Atto-565 and the actin cortex fluorescently tagged with LifeAct-Citrine at the equatorial plane of the T cells. (b) and (c) Corresponding merge image of the two images displayed in (a). (i) The membrane was located at the outer boundary (orange) and the cortex (green) at the inner boundary within the membrane-cortex interface of the left cell section; and the other way around in the right cell section (ii). Respective line profile revealed a total width of the membrane-cortex interface of  <500 nm (white arrows). Scale bars are 5 *µ*m and 500 nm, respectively.

Figure 3 displays representative dual-color STED images with the membrane located at the outer boundary and the cortical actin cytoskeleton located beneath the membrane. Additionally, the intra-cellular cytoplasmic actin cytoskeleton was visible, which interconnected with the cortical network (figures 3(a)–(c)). Plotting of the fluorescence intensity profile normal to the membrane-cortex interface revealed the membrane and actin cortex density distributions *ρ*_norm_ (see section 2). While the cortex density distribution (green) revealed a more complex shape with a finite width above 200 nm, the width of the membrane density distribution only reported on the spatial resolution of the STED microscope (~60 nm), as anticipated considering the finite thickness of the membrane of  <10 nm [35].

**Figure 4.**
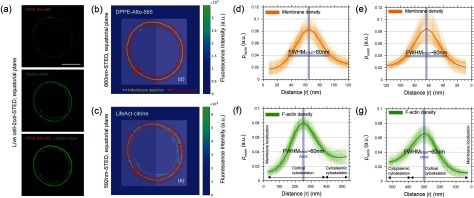
Computation of the membrane and actin cortex density distributions at the equatorial plane of live Jurkat T-cells. (a) Representative STED image of a Jurkat T-cell showing the plasma membrane fluorescently labeled with DPPE lipids (orange) tagged with Atto-565 (top panel) and the actin cortex fluorescently labeled F-actin (green) tagged with LifeAct-Citrine (middle panel), as well as the merged image of the plasma membrane and actin cortex (bottom panel). Scale bar is 5 *µ*m. (b) and (c) Pseudo-color image displays the detection of the membrane (dashed white line, (b)) and of the cortex (c) as well as the corresponding normal vectors in the direction of the calculation of the membrane and cortex profiles. (d) and (e) Membrane density distributions for the left (d) and the right (e) part of the cell. (f) and (g) Cortical actin density distributions for the left (f) and right (g) part of the cell showing asymmetric profiles with the cortical actin cytoskeleton interconnecting to the cytoplasmic actin network.

We used a custom-developed analysis algorithm for automated detection of the membrane, and computation of the membrane and cortex density distributions normal to the cell boundary (see section 2) (figures 4(a)–(c)). Figures 4(d) and (e) shows the average density distribution of the membrane including all individual measurements displayed for a representative T cell. All membrane densities were symmetric and revealed a finite width of 60 nm  ±  5 nm, which reports on the spatial resolution of the STED microscope, close to the one determined from the images of fluorescent beads (figure 2). Note, density distributions were normalized to their integral surface while intensity profiles were normalized to their maximum peak value (figure 3).

In contrast, the images of the actin filaments revealed an asymmetric density distribution pointing inwards in the direction of the cytoplasmic actin networks, with a mean width of 230 (+105/−  125) nm (figures 4(f) and (g)). As expected, this asymmetry of the density profile suggests inhomogeneous polymerization activity and thus a heterogeneous distribution of the dense actin filaments, spanning from the cortical actin cytoskeleton close to the membrane to the cytoplasmic actin networks. Notably, the peak positions of the density distributions of the membrane and actin cortex differ, with the peak shifted by multiple nanometers inwards in the case of the actin cortex, pointing to the nanoscale spacing of the membrane-cortex interface (as detailed further below).

**Figure 5.**
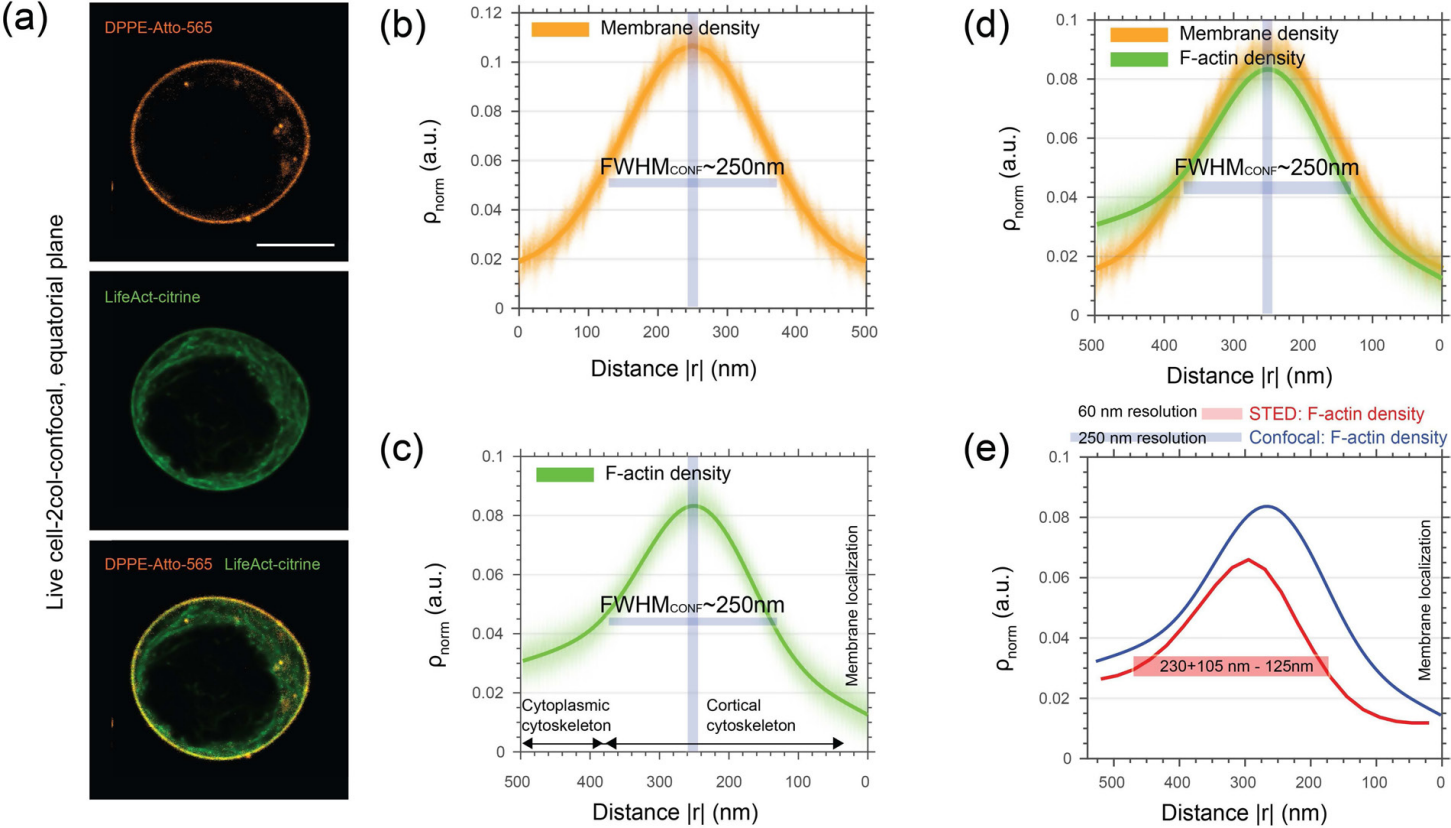
Confocal measurements of the membrane and actin cortex density distributions at the equatorial plane of live Jurkat T-cells. (a) Representative confocal image of a Jurkat T-cell showing the plasma membrane fluorescently labeled with DPPE lipids (orange) tagged with Atto-565 (top panel) and the actin cortex fluorescently labeled F-actin (green) tagged with LifeAct-Citrine (middle panel), as well as the merged image of the plasma membrane and actin cortex (bottom panel). Scale bars are 5 *µ*m. (b) Membrane density distributions for the the right part of the cell acquired by confocal. (c) Cortical actin density distributions for the right part of the cell acquired by confocal. (d) Overlay of actin and membrane density as shown in (b) and (c). (e) Comparing actin density distributions acquired by confocal and STED. Error bars show stds.

Let us briefly comment on the importance of employing the increased spatial resolution as provided by the STED microscope. Figure 5 display the density distributions of the actin cortex and the plasma membrane in live Jurkat T-cells as determined from images acquired by conventional confocal microscopy (figures 5(a) and (b)). Similar to the STED microscopy measurements, the membrane profile reveals the point-spread-function of the confocal microscope with its diffraction-limited resolution of ~250 nm (instead of 60 nm in the case of the STED microscope) (figures 5(b)–(d)). In the case of the actin cortex the limited spatial resolution of confocal microscopy did not allow to resolve the essential details of the actin density such as its asymmetry and its distance to the membrane. Specifically, the actin density was symmetric at the location of its maximum peak and then appeared to monotonically decay at the interface to the cytoplasm (in contrast to the overall highly asymmetric distribution extracted from the STED microscopy images) (figure 5(e)). Additionally, we could not observe a significant shift between the peaks of both (membrane and actin cortex) density distributions, precluding an accurate computation of the spatial distance between the cortex and the membrane.

### 3.3. Quantification of the nanoscale spacing between the membrane and actin cortex

**Figure 6.**
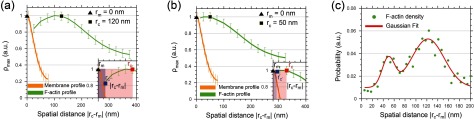
Nano-scale spacing between cortex and membrane at the equatorial plane of live Jurkat T-cells. (a) Membrane and cortex profiles as a function of the spatial distance for large distances. Zoom: nanoscale spacing was computed between the maximum peaks of the density distributions of the membrane (black triangle) and cortex (red square). (b) Membrane and cortex profiles as a function of the spatial distance for small distances. Zoom: nanoscale spacing was computed between the maximum peak of the membrane (black triangle) and outer boundary of the cortex (blue square). (c) Probability distribution averaged over all cells showing two distinct peaks for the spatial distances of the membrane and the actin cortex. Error bars show stds.

At this point, we calculated the spatial distance between the membrane and the cortex layer. We started by computing the spatial distance $|{{r}_{\text{c}}}-{{r}_{\text{m}}}|$ between the location of the maximum peak of the membrane density *r*_m_ and the location of the maximum peak of the cortex density *r*_c_ (figures 6(a) and (b); Zoom red region). Surprisingly, we discovered two scenarios within the membrane-cortex interface within each T cell. Sampling over all cells resulted in a bi-modal probability distribution of the spatial distance measured between the maximum density peaks of the cortex and the membrane, with mean values of 50  ±  15 nm and 120  ±  40 nm, respectively (figure 6(c)), suggesting some cortex regimes to be more tightly tethered to the membrane than others. Notably, the distances were not significantly different from those computed from dual color images acquired using the alternative labeling strategy (*p*  =  0.98 compared to alternative labeling strategy).

Finally, we computed the nanoscale spacing from the boundary location of the density distribution of cortical actin and the maximum peak location of the membrane (figure 6) instead of their maximum peak locations (see section 2). Taking into consideration the finite boundary of the cortical actin relative to the maximum peak of the membrane (figures 6(a) and (b); Zoom blue region), we found that the cortical actin was closer than 10 nm to the membrane in some regions and a maximum of 20 nm in others, suggesting two different types of interaction dynamics governing the membrane-cortex interface.

## 4. Concluding discussion and outlook

In this article, we accurately measured the density distribution of the actin cortex and its nanoscale spacing to the plasma membrane in live Jurkat T-cells using dual color super-resolution STED microscopy. We found an asymmetric density distribution of cortical actin beneath the membrane towards the cytoplasmic direction, indicating that actin is not homogeneously distributed throughout the cortex, consistent with recent analytical predictions from active-gel theory [11]. Remarkably, our experiments demonstrated that the actin density was not maximal at the membrane boundary as it could be expected from earlier work [8] but occurred at a finite distance from the membrane boundary with a distinct maximum and the aforementioned wider and flatter decay towards the cytoplasmic actin network. Inhomogeneous actin densities could be caused by changes in the number of actin filaments throughout the cortex or non-isotropic actin orientations. However, the latter would contradict recent observations of isotropic actin filament orientations in the cortex [9]. Alternatively, different actin filaments lengths and thus actin filament families might be present throughout the cortex such as short-length Arp2/3-nucleated and long-length formin-nucleated actin filaments [9, 36]. This notion is also supported by the fact that formin-nucleated actin, which is 20 times less abundant than Arp2/3-nucleated F-actin, is predominantly generated at the membrane boundary, while Arp2/3-nucleated F-actin is preferentially created by binding to pre-existing F-actin [24, 37].

We also measured the nanoscale spacing between the membrane and the actin cortex and found a bi-modal distribution (even within one Tcell), reporting on high and low regimes of membrane-cortex adhesion. Notably, the bi-modal distribution of the nanoscale spacing was independent from the computation reference system. In both computation scenarios, the spatial distances, computed either between the maximum peak values of membrane and cortex or between the maximum peak value of the membrane and the outer cortex boundary, revealed two regimes of membrane-cortex adhesion. A previous attempt to determine the nanoscale spacing from electron tomography reconstructions in PtK2 cells was consistent with our data [16], but missed essential details such as the regimes of low adhesion energy.

Neither the actin-cortex density nor the distance between the membrane and actin cortex could accurately be determined from dual-color confocal images. Due to the limited spatial resolution of the confocal microscope (of about 250 nm compared to the 50–60 nm of the employed STED microscope), the actin density appeared almost symmetrically distributed and there was no notable shift observed between the peaks of the density distributions of the membrane and actin cortex. This difference between the STED and confocal recordings also highlights the advancements compared to previous confocal monitoring of the actin cortex thickness [8]. To determine values of the thickness, a uniform actin density and a direct contact between the cortical layer and the plasma membrane had to be assumed. Yet, one has to note that those measurements were performed on HeLa cells, while our measurements were done on T cells, and we cannot exclude possible differences in actin cortex density and membrane-cortex distance between both cell types.

Cells can locally or globally increase the adhesion energy between the membrane and the actin cortex by, for example, over-activating ERM proteins and in this way regulate their morphology, mechanics, and function [5, 20, 21]. On the other hand, the tighter and looser regimes might report on the polymerization state of the actin cortex. In fact, the steady state density distributions of cortical actin has been predicted from active gel theory to differ in regimes of weakly and strongly active polymerization [11]. Specifically, regimes of increased net-actin polymerization activity would result in high actin abundance and therefore locally increase the probability for ERM protein binding and thus membrane-cortex tethering. In turn, low actin abundance at the membrane boundary would kinetically hamper ERM binding and thus result in weaker membrane-cortex coupling.

From a mechanical point of view, the tight membrane-cortex coupling would explain the many effects of cortical actin on the membrane architecture such as ligand receptor organization and dynamics, as outlined in the introduction. The ability to dynamically adjust the spacing between the cortical actin and the membrane allows cells to tune membrane architecture including protein and lipid distribution, aggregation, and their diffusion-reaction dynamics throughout the lipid membrane bilayer and thereby to regulate e.g. cellular signaling events. Moreover, one may speculate that cells actively use principles of actin assembly or self-organization to reshape cortical actin into patterns such as asters, and in this way to reorganize and regulate membranous protein arrangements, as it has been predicted previously [38].

Taken together, this work focused on the steady state distribution of the actin cortex and its relationship to the cellular plasma membrane in living cells. We restricted our measurements to the geometric center of the cells and therefore the results only report on those cortical density distributions and nanoscale spacing at the equatorial plane, which may strongly vary from the apical and, in particular, the basal densities, where the cells are interacting with a solid surface. In the future, improvements in the axial spatial resolution of the STED microscope may allow experimental measurements at those missed locations as well. We believe, however, that our measurements of the cortical actin density and the spatial distance between membrane and actin cortex provide new insights into the importance of the membrane-cortex interface and its implications on the membrane architecture and function.
